# Studies on the High-Power Piezoelectric Property Measurement Methods and Decoupling the Power and Temperature Effects on PZT-5H

**DOI:** 10.3390/s25020349

**Published:** 2025-01-09

**Authors:** Wenchao Xue, Xiaobo Wang, Yuliang Zhu, Chengtao Luo

**Affiliations:** School of Electronic Information and Electrical Engineering, Shanghai Jiao Tong University, Shanghai 200240, China; wenchao.xue@sjtu.edu.cn (W.X.); 022035910015@sjtu.edu.cn (X.W.); zhuyl2@sjtu.edu.cn (Y.Z.)

**Keywords:** high-power characterization, piezoelectric transducer, piezoelectric ceramic, material parameter, transient method

## Abstract

For those piezoelectric materials that operate under high-power conditions, the piezoelectric and dielectric properties obtained under small signal conditions cannot be directly applied to high-power transducers. There are three mainstream high-power characterization methods: the constant voltage method, the constant current method, and the transient method. In this study, we developed and verified a combined impedance method that integrated the advantages of the constant voltage and current methods, along with an improved transient method, for high-power testing of PZT-5H piezoelectric ceramics. The results from both methods indicated that with increasing power, the electromechanical coupling coefficient $k_{31}$ , the piezoelectric constant $d_{31}$, and the elastic compliance $s_{11}^{E}$ of the PZT-5H showed increasing trends, while the mechanical quality factor $Q_{m}$ first decayed rapidly and then stabilized at a fixed level. Additionally, under the combined impedance method, the temperature of the vibrators rose significantly due to self-heating, whereas the transient method generated almost no heat, and the vibrators remained at room temperature. By comparing the results from the two methods, we decoupled the effects of temperature and power on the high-power piezoelectric performance. The results showed that the self-heating temperature amplified the effects of power on $k_{31}$, $d_{31}$, and $s_{11}^{E}$, while its influence on $Q_{m}$ remained negligible.

## 1. Introduction

High-power piezoelectric transducers have seen rapid development in recent years and are widely used in applications like sonar [1], ultrasonic welding [2], ultrasonic inspection [3], and acoustically driven antennae [4]. These transducers often pursue high performance, such as large bandwidths and good sensitivity. Thus, their core piezoelectric materials must possess high piezoelectric properties, such as the piezoelectric coefficients $d_{31}$ and the mechanical quality factors $Q_{m}$ [5]. Although various new piezoelectric materials have been developed for high-power transducers [6], it is difficult to apply the upgraded materials, as it is impractical to apply the existing small-signal measurement results directly to the high-power transducer design. Because many properties, including $d_{31}$ and $Q_{m}$, exhibit significant nonlinear changes under high-power conditions [7], it is crucial to measure the piezoelectric properties under high-power conditions, especially due to the demands of finite element methods for transducer design and simulation [8].

After decades of development, high-power characterization methods for piezoelectric materials now include constant voltage, constant current, and transient methods. However, the comparison of these methods on the same material has not yet been thoroughly studied, and thus, there are no widely accepted standards for high-power measurement yet. In addition, although it is already known that the piezoelectric properties are power- or voltage-dependent under high-power conditions, whether the origin of such dependence is due to the self-heating temperature effect or the high-power vibration velocity effect alone is still unclear. Currently, none of the previously mentioned methods alone can decouple these two effects on the high-power piezoelectric property measurement.

Both the constant voltage method [9] and the constant current method [10] belong to the impedance spectroscopy method. These methods directly measure the impedance spectrum under the constant voltage or constant current conditions, providing the power-dependent resonant and anti-resonant frequency. These methods are simple to operate but also have unignorable disadvantages. The impedance spectra from the constant voltage method are distorted near the resonant frequency $f_{s}$ and show obvious spectral hysteresis, which hinders the precise calculations for piezoelectric transducer design [11]. The mechanism underlying this phenomenon remains unclear. Slabki et al. attribute it to the large electric fields and mechanical stresses causing non-linear material properties [12], while Uchino et al. suggest that it is due to the nonlinear behavior of elastic compliance at high-vibration amplitudes [13]. It is worth noting that the resonant frequency and anti-resonant frequency mentioned in this paper actually refer to the series resonant frequency ($f_{s}$) and the parallel resonant frequency ($f_{p}$), which are electrically defined based on impedance variations and are approximately equal to the resonant frequency ($f_{r}$) and anti-resonant frequency ($f_{a}$) [14]. On the other hand, the constant current method requires unrealistically high-voltage to measure the anti-resonant frequency. Such demands on the equipment are unacceptable because the impedance of the tested sample at the anti-resonant frequency $f_{p}$ is usually 10 to 100 times higher than that at the $f_{s}$ [15]. In response to these challenges, Uchino et al. proposed that the $f_{s}$ can be obtained using the constant current method, while the $f_{p}$ can be determined using the constant voltage method [16]. However, the feasibility of such a method combination is unknown, and the operation standards have not been fully discussed. Therefore, we developed the combined impedance method by setting standardized and practical rules in this work. This method integrates the advantages of the constant voltage and current methods, making it possible to obtain the high-power impedance spectrum more accurately.

The transient method [17] also provides the resonant frequency of the vibrator. It does not measure the impedance spectra directly but measures the vibrators’ decay curves of vibration velocity and current after the transient excitation. This method first applies the sinusoidal pulse voltage signal near the resonant frequency to the piezoelectric vibrator. After the vibrator is thoroughly excited, the circuit is shortened, switching the vibrator to its resonant state. The decaying process of the vibration is then recorded and can be used to calculate the piezoelectric properties. This method offers many advantages, such as short testing times, minimal heat generation, and, most importantly, the decoupling of the parameters from the temperature effects. Some approaches suggest that the decaying process exhibits an exponential curve, using a constant decay coefficient during the fitting. Umeda and others pointed out that the decay coefficient β of vibration velocity depends on the vibration velocity itself [18]. Thus, they perform segmented fitting on a single decay process to derive β and $Q_{m}$ at different vibration velocities or power levels. Currently, modeling and analysis of the decaying process of vibration are still being developed.

In this paper, we proposed a new method for testing piezoelectric vibrators: the combined impedance method. Then, we demonstrated the feasibility and rationality of this method and established its operational standards. We employed the combined impedance and transient methods for the high-power measurement of PZT-5H piezoelectric ceramics. Using these methods, we obtained high-power material parameters, including the $k_{31}$, $d_{31}$, $s_{11}^{E},$ and $Q_{m},$ and observed significant nonlinear phenomena. Additionally, due to the different self-heating temperatures associated with each method, this study compared material parameters at the same power levels but at different self-heating temperatures. This led to insights into the impacts of self-heating temperatures on the nonlinearity of piezoelectric materials.

## 2. Materials and Methods

The material of the piezoelectric vibrators tested in this paper was PZT-5H ceramic. Due to its low $Q_{m}$, it heats up easily under high power, making it suitable for studying the temperature dependence of the vibrators’ properties. The dimension of each piezoelectric vibrator was 30 × 8 × 0.5 mm, which complies with the IEEE standard for transverse vibrational modes [19]. The material properties of PZT-5H are listed in Table A1 in Appendix A. In the experiment, two steel needles were clamped at the geometric center of the vibrator, making it vibrate horizontally in the free-boundary conditions and providing electrical connections. Both the combined impedance and the transient methods utilized a Doppler laser vibrometer (Polytec OFV 505, Polytec, Waldbronn, Germany) for real-time vibration velocity measurements. An infrared thermal imager (Fotric 246M, Fotric, Shanghai, China) was used for the real-time surface temperature measurements of the vibrator.

### 2.1. Combined Impedance Method

The combined impedance method described in this paper combined two impedance spectra ($\left|Z\right|-f$) measured separately by the constant current method and the constant voltage method. Two spectra were combined at a combination point. At this point, the vibrators under the constant current and the constant voltage methods had the same vibration velocity, guaranteed by the Doppler laser vibrometer.

**Figure 1:**
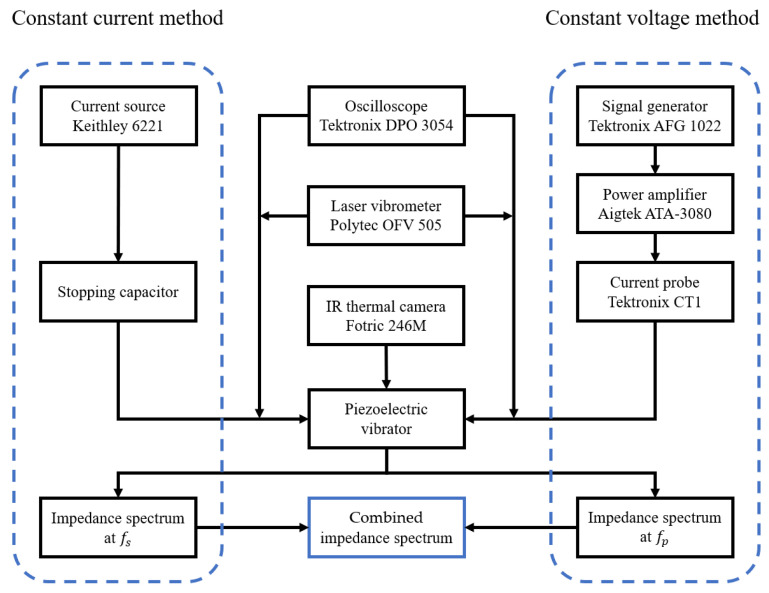
Schematic diagram of the combined impedance method.

The schematic diagram of the combined impedance method is shown in Figure 1. The constant current method used a constant current source (Keithley 6221, Keithley, Cleveland, OH, USA), and the spectrum near the $f_{s}$ was recorded by an oscilloscope (Tektronix DPO 3054, Tektronix, Beaverton, OR, USA). The constant voltage method used the signal generator (Tektronix AFG1022, Tektronix, Beaverton, OR, USA) and power amplifier (Aigtek ATA 3080, Aigtek, Xi’an, China) as the constant voltage source. An additional current probe (Tektronix CT1, Tektronix, Beaverton, OR, USA) was used to record the spectrum near the $f_{p}$. The location of the combination point was set where the impedance was 1.6 times the resonance impedance at maximum power and where the frequency was between the $f_{s}$ and $f_{p}$. This rule ensured that the voltage applied by the constant voltage method remained within a reasonable range. Also, it was essential to provide a constant ratio (about 250 Ω in this work) between the voltage used in the constant voltage method and the current used in the constant current method. This made it more reasonable to determine the voltage values and made the combined $\left|Z\right|-f$ spectra comparable in the form of power.

Using the impedance spectra obtained from the combined impedance method, various parameters of the piezoelectric vibrator can be calculated [20]:

(1) $$\begin{array}{c} k_{31}^{2}/\left(1-k_{31}^{2}\right)=\left(\pi /2\right)\left(f_{p}/f_{s}\right)\tan\left[\left(\pi /2\right)\left(f_{p}-f_{s}\right)/f_{s}\right] \end{array}$$

(2) $$\begin{array}{c} s_{11}^{E}=1/\left(4\rho x^{2}f_{s}^{2}\right) \end{array}$$

(3) $$\begin{array}{c} d_{31}=k_{31}\sqrt{\varepsilon_{33}^{T}s_{11}^{E}} \end{array}$$

where ρ is the density of the piezoelectric vibrator, x is the length of the piezoelectric vibrator, and $\varepsilon_{33}^{T}$ is the permittivity.

When the piezoelectric vibrator is in a resonant state, it can be equivalently represented as an inductor $L_{1}$, capacitor $C_{1}$, and resistor $R_{1}$ in series, which are then paralleled with $C_{0}$ [20]:

(4) $$\begin{array}{c} C_{1}=\frac{8xyd_{31}^{2}}{\pi^{2}zs_{11}^{E}} \end{array}$$

(5) $$\begin{array}{c} L_{1}=\frac{\rho xz}{8y}{\left(\frac{s_{11}^{E}}{d_{31}}\right)}^{2} \end{array}$$

(6) $$\begin{array}{c} C_{0}=\frac{xy}{z}\varepsilon_{33}^{T}\left(1-k_{31}^{2}\right) \end{array}$$

(7) $$\begin{array}{c} R_{1}=\frac{{\left|Z\right|}_{min}}{\sqrt{1-\omega_{s}^{2}C_{0}^{2}{\left|Z\right|}_{min}^{2}}} \end{array}$$

(8) $$\begin{array}{c} Q_{m}=\frac{1}{R_{1}}\sqrt{\frac{L_{1}}{C_{1}}} \end{array}$$

where y is the width of the piezoelectric vibrator, z is the thickness of the piezoelectric vibrator, $\omega_{s\;}$ is the resonant angular frequency, and ${\left|Z\right|}_{min}$ is the minimum impedance of the piezoelectric vibrator at the resonant frequency.

### 2.2. Transient Method

**Figure 2:**
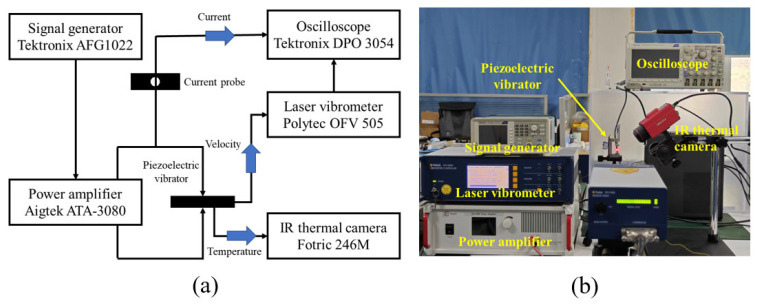
(**a**) Schematic diagram of the transient method and (**b**) experimental setup of the transient method.

**Figure A1:**
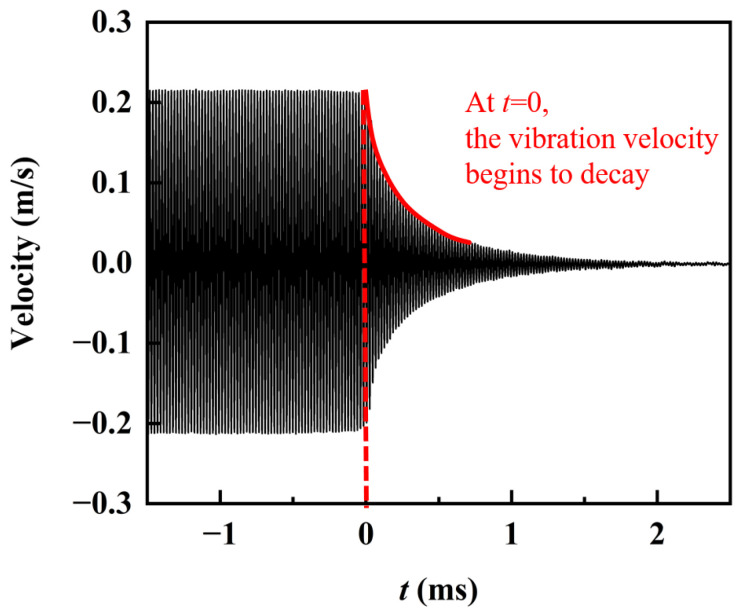
Decay curve of the vibration velocity of the piezoelectric vibrator (5 Vpp).

The principle of the transient method and the data flow direction are illustrated in Figure 2a, and the experimental setup is shown in Figure 2b. During the test, the vibrator was first fully excited by a short sinusoidal pulse (about 2 ms), which was provided by a signal generator (Tektronix AFG1022, Tektronix, Beaverton, OR, USA) and a power amplifier (Aigtek ATA 3080, Aigtek, Xi’an, China). Then, the vibrator was shortened at the time marked as t = 0, and the vibration began to decay in its resonant state. A Doppler laser vibrometer (Polytec OFV 505, Polytec, Waldbronn, Germany) and a current probe (Tektronix CT1, Tektronix, Beaverton, OR, USA) were used to measure the decay curves of the vibration velocity and the current simultaneously. Finally, we used a low-pass filter to eliminate the higher-order harmonics, and then the decay curves (t>0) can finally be described as Equation (9) below:

(9) $$\begin{array}{c} V=V_{0}e^{-\beta t}\sin2\pi f_{s}t \end{array}$$

where $V_{0}$ is the amplitude of the vibrator’s velocity at t = 0, $f_{s\;}$ is the resonant frequency, and β is the decay coefficient of the velocity decay curves where this coefficient changes over time and is not constant. An example of the decay curve corresponding to the 5 V is provided in Figure A1 in Appendix A.

The β in Equation (9) is essential because some piezoelectric parameters of the vibrator, $Q_{m},$ and $d_{31}$, are related as follows [19]:

(10) $$\begin{array}{c} Q_{m}=\frac{\pi f_{s}}{\beta } \end{array}$$

(11) $$\begin{array}{c} d_{31}=\frac{s_{11}^{E}I}{2yV} \end{array}$$

In the equations above, I is the amplitude of the current through the vibrator, and V is the amplitude of the vibration velocity. It is noteworthy that the formulas for calculating $s_{11}^{E}$ and $k_{31}$ in the transient method are identical to those used in the combined impedance method, as shown in Equations (1) and (2).

The development of fitting methods for β can be divided into two stages. The earliest approach involved directly fitting the decay curve to obtain a fixed β. However, this method results in significant errors, because β continuously changes during the decay process. In the second stage, a segmented fitting method was introduced. This method fits multiple $\beta_{i}$ at different amplitudes $V_{i}$ on the same decay curve and calculates the $Q_{m}$ for each amplitude using the corresponding single power and resonant frequency of the decay curve. While this approach reflects the variation of β with $V_{i}$, it has its limitations. The transient amplitude $V_{i}$ during the decay process corresponds to a different power as compared to the steady-state $V_{i}$, and the resonant frequency changes with power. Therefore, using a single resonant frequency and power to calculate all $Q_{m}$ is not reasonable. To address this issue, we applied different powers to achieve various steady-state amplitudes $V_{i}$ and obtained corresponding decay curves. By calculating $\beta_{i}$ at each amplitude $V_{i}$, we derived the $Q_{m}\;$ under different power conditions, thereby minimizing potential errors to the greatest extent possible. Here, $V_{i}$ is only related to the applied power, and the same lengths of the decay curves from the beginning (three cycles from t=0, approximately (62.5 μs) are cut for the curve fitting. Because the time length is sufficiently short, it can be assumed that the β is nearly constant for the cut curves, thereby calculating a more accurate $\beta (V_{i})$ or β(P). This approach enhances the consistency of β measurement at different power levels.

## 3. Results and Discussion

### 3.1. The Feasibility of the Combined Impedance Method

**Figure 3:**
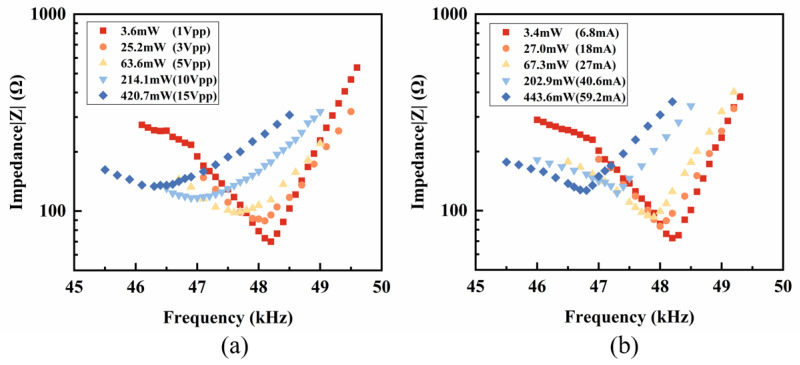
(**a**) Impedance spectra at resonance under the constant voltage method at different powers and (**b**) impedance spectra at resonance under the constant current method at different powers.

**Figure A2:**
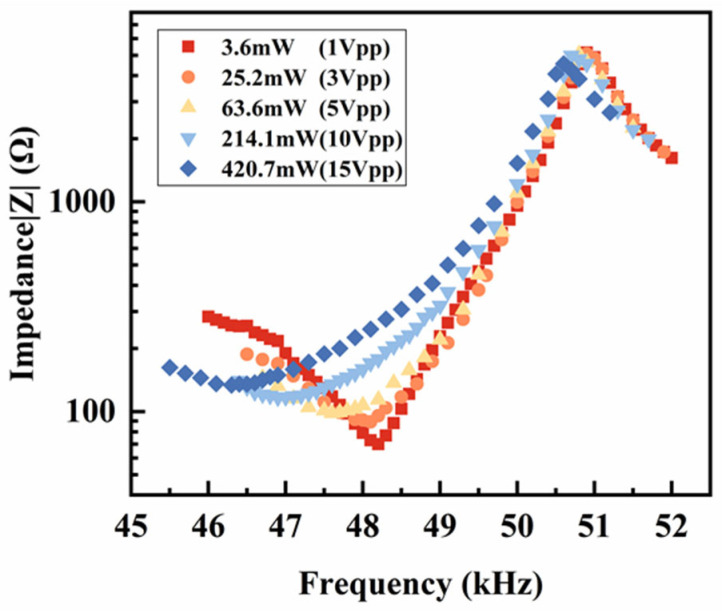
Impedance spectra measured using the constant voltage method.

To verify the necessity and rationality of the combined impedance method, we first measured the spectra of the PZT-5H vibrator, using the traditional constant voltage and constant current methods separately. The complete spectra obtained from the constant voltage method are shown in Figure A2 in Appendix A. First, the constant voltage method was proven more suitable for measuring the impedance spectrum near the $f_{p}$. Due to the high impedance near the $f_{p}$, the power output requirement for the constant current method is too high to maintain. When measuring the impedance spectrum near the $f_{s}$, both the constant voltage method (shown in Figure 3a) and the constant current method (shown in Figure 3b) show the significant shifting of the $f_{s}$ with the increasing power. However, the impedance peaks at the $f_{s}$ and the frequency shifting of the $f_{s}$ measured by the constant current is less distorted in the high-power conditions. Therefore, the constant current method was found to be more suitable for these measurements. These conclusions agree with the study by Uchino et al. [16] and prove that it is essential to develop a combined impedance method that can integrate the advantages of constant voltage and constant current methods. However, there are no widely accepted and practical operation standards for such a combination yet.

### 3.2. The Standards of the Combined Impedance Method

**Figure 4:**
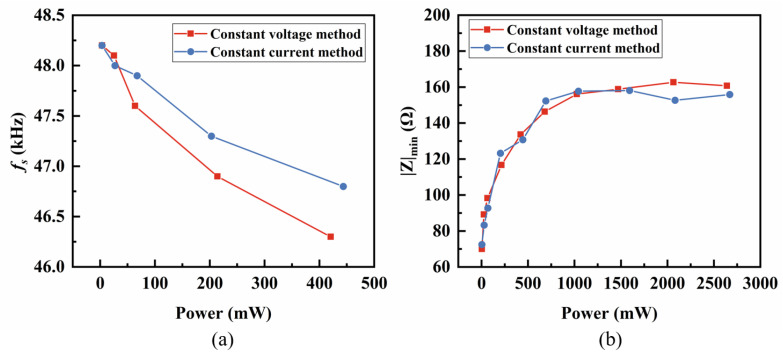
(**a**) Comparison of the $f_{s}$ between the constant current method and the constant voltage method and (**b**) comparison of the impedance at the $f_{s}$ between the constant current method and the constant voltage method.

**Figure 5:**
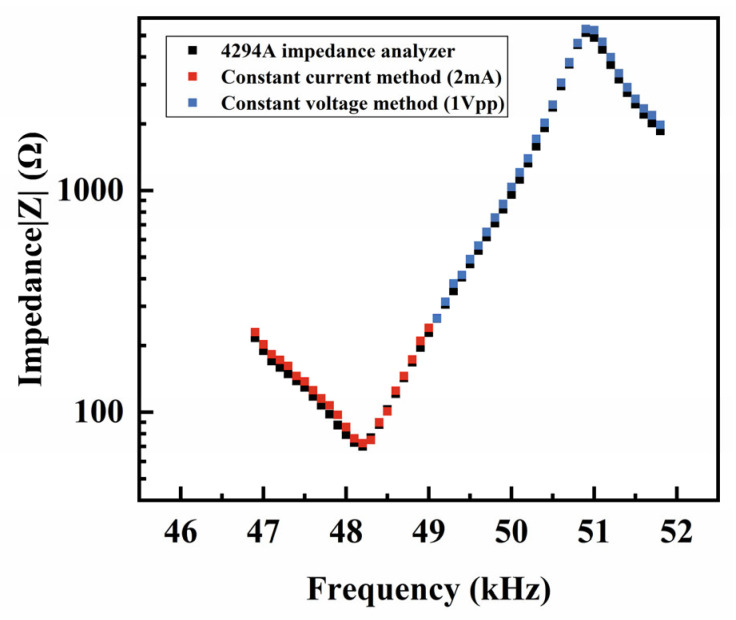
Comparison of impedance spectra among the combined impedance method and 4294 impedance analyzers under the small-signal condition (1 Vpp).

**Figure A3:**
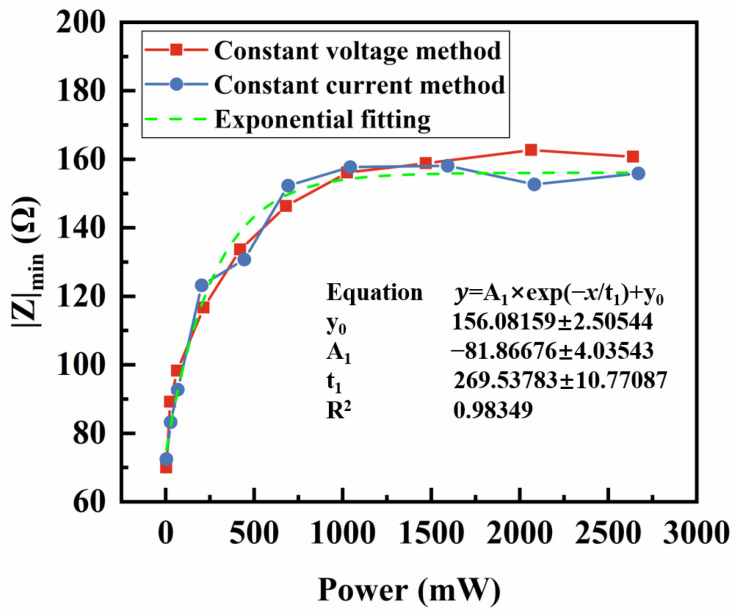
Exponential fitting of ${\left|Z\right|}^{*}$ in the constant current method.

Consequently, we proposed a viable approach to determine the location of the combination point by combining two spectra, as shown below. First, Figure 4 compares the $f_{s}$ and the impedance at the $f_{s}$ from both the constant current and constant voltage methods. Figure 4a reveals that the $f_{s}$ shifting is approximately negatively correlated to the applied power, while the impedance at $f_{s}$ (${\left|Z\right|}_{min}$) becomes saturated at a fixed value ${\left|Z\right|}^{*}$ with the increasing applied power. This conclusion is also supported by the relevant literature [20]. We accurately determined ${\left|Z\right|}^{*}$ in the constant current method using exponential fitting, with the fitting results shown in Figure A3 in Appendix A. In this experiment, ${\left|Z\right|}^{*}$ was determined to be 156 Ω through curve fitting. Next, we selected a fixed value ${\left|Z\right|}_{c}>{\left|Z\right|}^{*}$ as the combination point. We chose to set the impedance value of the combination point ${\left|Z\right|}_{c}$ as a fixed value, mainly to ensure that the voltage applied near the anti-resonance region of the impedance spectrum at different powers is proportional to the current applied in the constant current method. The combination point impedance should not be too close to ${\left|Z\right|}^{*}$, as the high-vibration velocity at resonance would make it difficult to maintain a consistent vibration speed when switching from the constant current method to the constant voltage method, complicating experimental operations. Conversely, if the combination point impedance is significantly higher than ${\left|Z\right|}^{*}$, it may result in the vibration velocity of the piezoelectric vibrator becoming too low to be accurately measured. Therefore, we initially defined the range of the combination point impedance as ${{\left|Z\right|}^{*}<|Z|}_{c}<2*{\left|Z\right|}^{*}$. After multiple experimental trials and a comprehensive consideration of the experimental margins and the accuracy of ${\left|Z\right|}^{*}$, we ultimately adjusted the combination point impedance to ${\left|Z\right|}_{c}=1.6{\left|Z\right|}^{*}$. In this experiment, the final impedance value of the combination point was set to 250 Ω. Ultimately, the combined spectrum was measured under the small-signal condition to test its compatibility with the spectrum from the traditional impedance analyzer (Agilent 4294A, Agilent, Santa Clara, CA, USA). As shown in Figure 5, the combined impedance method here uses the constant current of 4 mA peak-to-peak and the constant voltage of 1 Vpp. Two spectra here closely match each other, and thus, the accuracy of the combined impedance method is proven under the small-signal condition.

### 3.3. The Comparison Between the Two Methods

**Figure 6:**
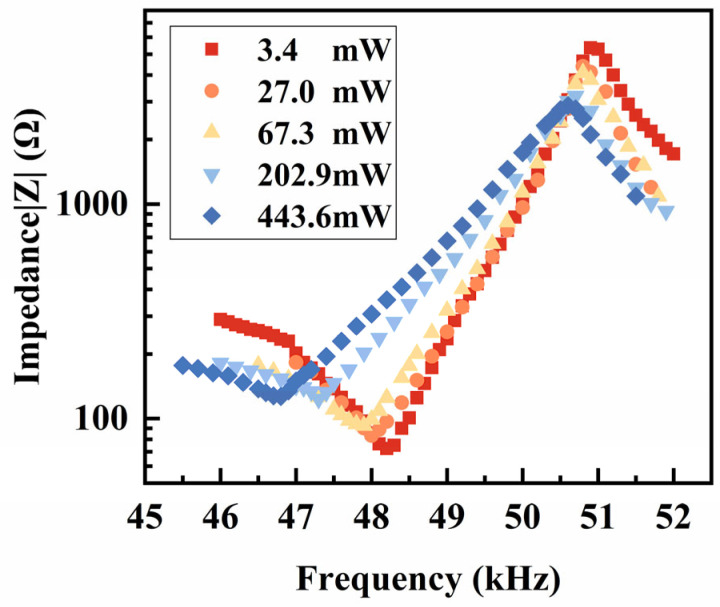
Impedance spectra measured using the combined impedance method.

After verifying the feasibility and setting the standards of the combined impedance method, we used this method to measure the impedance spectra under the different power levels. The results are illustrated in Figure 6, and the spectra are labeled by the applied power at the $f_{s}$. Figure 6 provides the power-dependent $f_{s}$ and $f_{p}$, which are further discussed later in this chapter.

**Figure 7:**
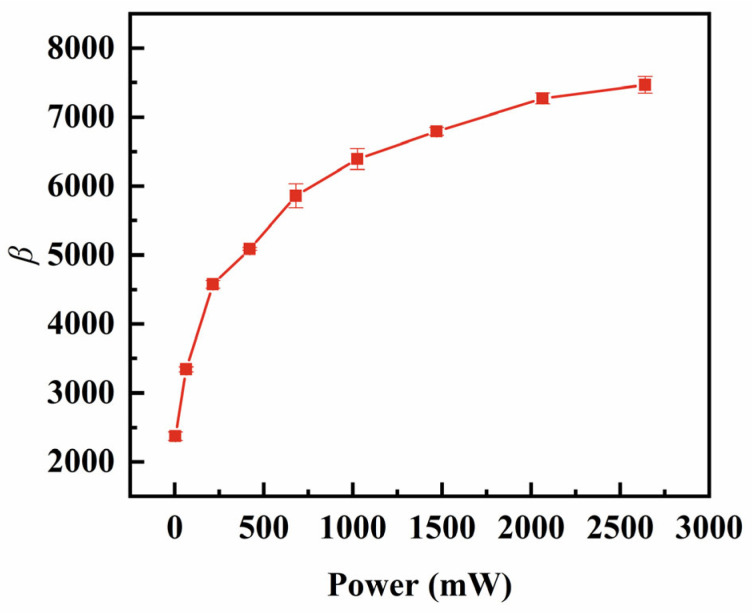
Curve of the vibration velocity decay coefficient versus power.

**Figure 8:**
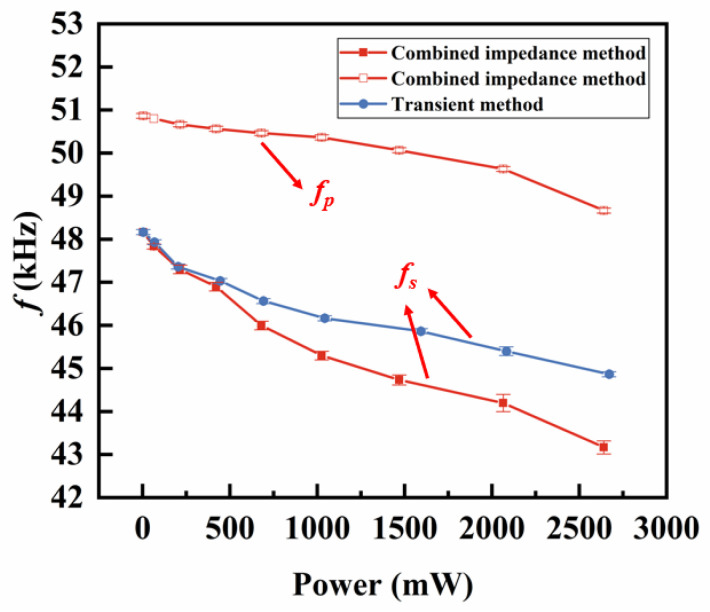
$f_{s}$ and $f_{p}$ of the combined impedance method and the transient method.

On the other hand, we used the transient method to measure the decay curves under the different power conditions. The transient method tuned the power through the applied pulse amplitude. The power dependence of the decay coefficient β was first measured as shown in Figure 7. The β initially increases rapidly and then stabilizes with growing power. Meanwhile, the power dependence of the $f_{s}$ measured from the transient method is compared with the $f_{s}$ and $f_{p}$ measured from the combined impedance method in Figure 8. The results from the two methods are compared through the applied power at the $f_{s}$.

**Figure 9:**
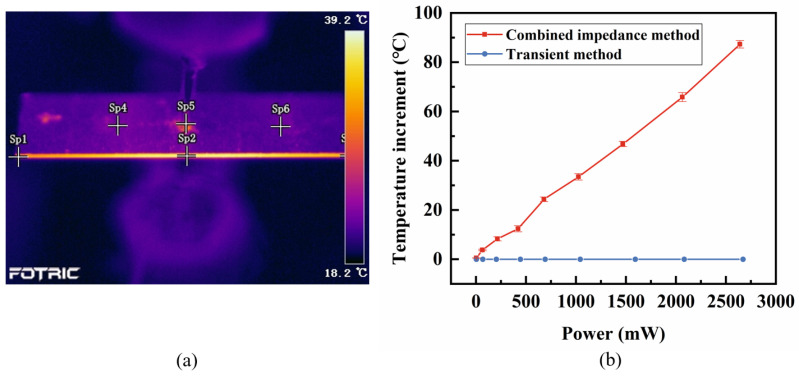
(**a**) Temperature distribution measured by the infrared thermal imager and (**b**) temperature comparison between the combined impedance method and the transient method.

Figure 8 shows that as the power increased, $f_{s}$ and $f_{p}$ as measured by both methods exhibit similar decreasing trends, while the decline rate of the $f_{s}$ is significantly faster than that of the $f_{p}$ in the figure. Additionally, at low powers (<250 mW), the $f_{s}$ measured by the transient method is close to that measured by the combined impedance method. As the power increases, the $∆f_{s}$ between the two become more pronounced. The increase of $∆f_{s}$ is primarily due to the temperature effects because it is the only difference between these two methods. The combined impedance method involves a lengthy testing process that generates significant self-heating, causing the temperature of the piezoelectric vibrator to rise. In contrast, the transient method has a very short testing duration that hardly changes the temperature. These temperature effects are quantitively proven by the temperature measurement results shown in Figure 9. Additionally, the temperature rising due to the self-heating under the free boundary condition is found to be linearly correlated to the applied power.

### 3.4. Parameter Comparison

**Figure 10:**
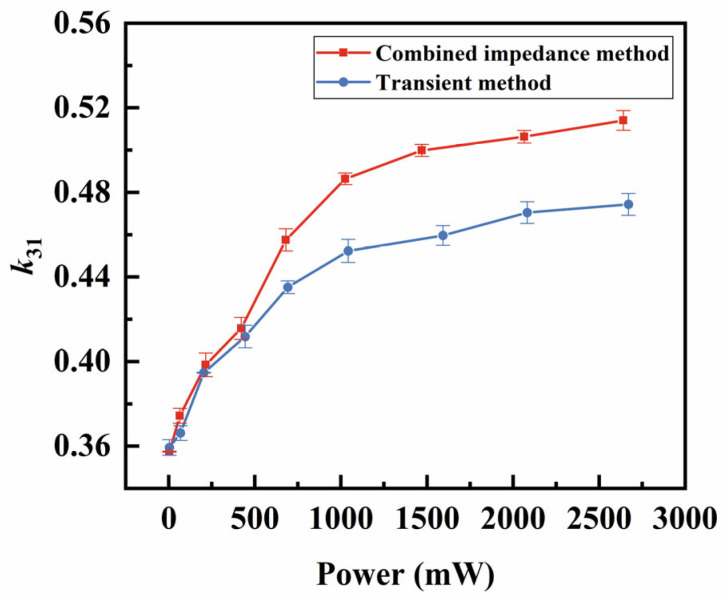
Comparison of the $k_{31}$ between the combined impedance method and the transient method.

**Figure 11:**
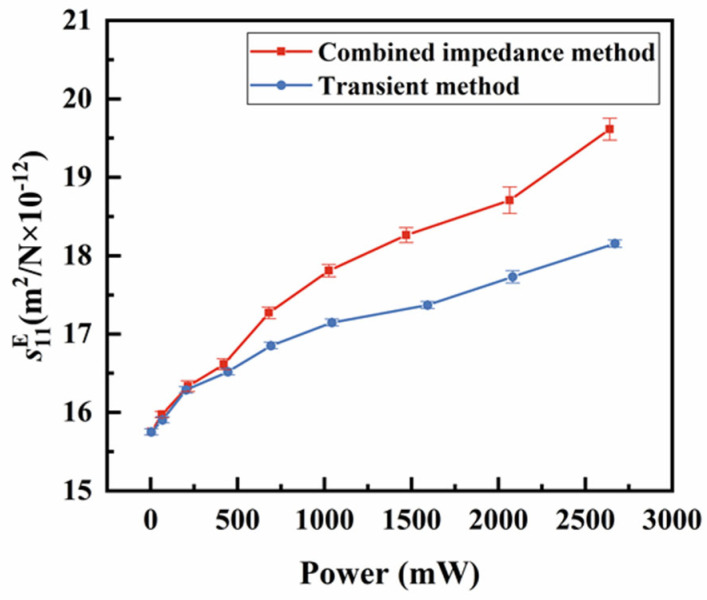
Comparison of the $s_{11}^{E}$ between the combined impedance method and the transient method.

**Figure 12:**
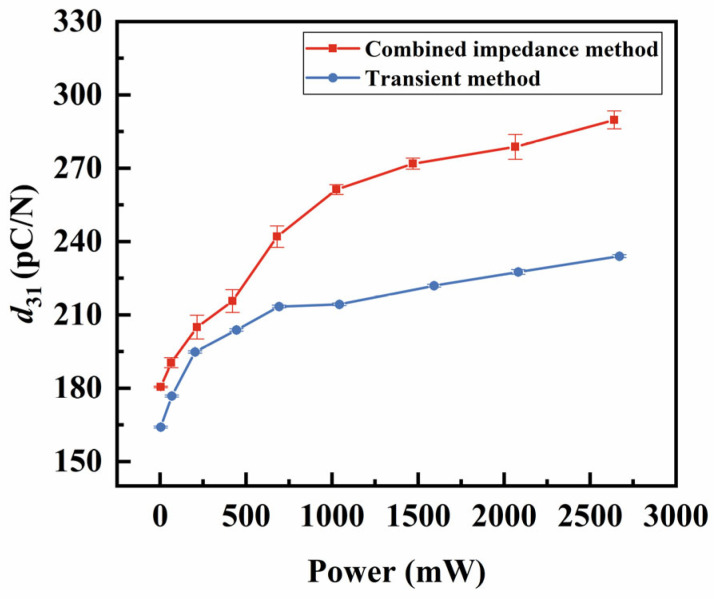
Comparison of the $d_{31}$ between the combined impedance method and the transient method.

In addition to the $f_{s}$, other temperatures’ effects on the vibrator’s properties were studied by comparing the parameters measured using the two methods. Previous studies have explored the effects of temperature on resonant frequency or piezoelectric parameters, primarily focusing on the increase in the ambient temperature or self-heating caused by elevated power levels [21,22]. However, our study uniquely employs a comparative approach between two methods to isolate the effect of self-heating temperature, independent of power or voltage influences, providing a more focused and insightful analysis. The electromechanical coupling coefficient $k_{31}$ measures the effectiveness of energy conversion between the mechanical and electrical energy in a piezoelectric vibrator. As shown in Figure 10, as the power increases, the $k_{31}$ calculated by both methods initially rise quickly and then level off. The $∆k_{31}$ between the two methods is not significant until the power reaches about 420 mW, where the temperature difference ∆T between the two methods is 12 °C. At maximum power (about 2670 mW), this difference reaches approximately 90 °C, while the combined impedance method showing $k_{31}$ is 8% higher than that of the transient method. The parameter $s_{11}^{E}$, which characterizes the degree of the deformation of the piezoelectric materials under external force, is shown in Figure 11. As power increases, the measured value of $s_{11}^{E}$ gradually rises, with a higher rate of increase observed for the combined impedance method. This suggests that temperature contributes to an increase in $s_{11}^{E}$. The $d_{31}$ represents the relationship between the mechanical and electrical responses. Similar to $k_{31}$, the value of ${∆d}_{31}$ gradually increases when the applied power exceeds approximately 420 mW, as shown in Figure 12. By analyzing $k_{31}$, $d_{31}$, and $s_{11}^{E}$, we observed a similar trend: under the same power conditions, the parameters obtained from the two methods are relatively close at a low power (<420 mW), while the differences become more pronounced at a high power (>420 mW). Thus, we can exclude the influence of power and further confirm that the increase in the self-heating temperature promotes the enhancement of these three piezoelectric parameters.

**Figure 13:**
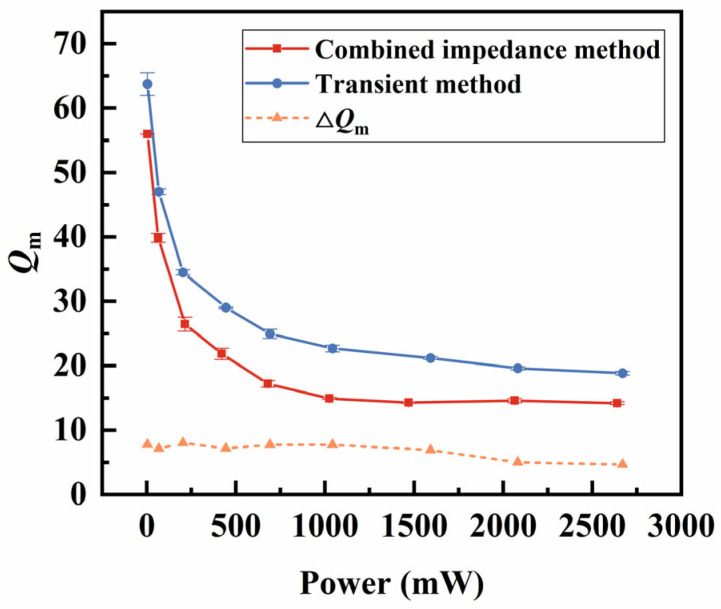
Comparison of $Q_{m}$ between the combined impedance method and transient method.

Finally, in high-power piezoelectric transducers, the $Q_{m}$ indicates the energy loss in the piezoelectric vibrators and is one of the key parameters determining the output power of piezoelectric transducers. Generally, a higher $Q_{m}$ indicates less energy loss during conversion, making it more suitable for high-power applications. As shown in Figure 13, the $Q_{m}$ calculated by both methods decrease rapidly with increasing power, and then above 1000 mW, their values tend to stabilize. With the combined impedance method, as the power increases, the temperature rises quickly, yet the $Q_{m}$ still tends to stabilize. Even though the transient method is decoupled from temperature, it still shows a rapid decline in $Q_{m}$. Under both low- and high-power conditions, the $Q_{m}$ calculated by the transient method is slightly higher than those calculated by the combined impedance method. As shown in Figure 9b, the temperature difference between the two methods increases from 0 °C to approximately 90 °C across the entire power range. By calculating the $∆Q_{m}$ between the two methods, we found that the difference remains relatively unchanged across the entire power range despite the increase in temperature. This indicates that temperature is not the main factor affecting the $Q_{m}$. Instead, it is related to the enhanced movement of the ferroelectric domain walls at high-vibration velocities [23].

## 4. Conclusions

This paper developed a new method for testing piezoelectric vibrators, termed the combined impedance method, which integrates the advantages of the constant voltage and current methods. Then we demonstrated the feasibility and rationality of this method and established its operational standards. Additionally, we improved the transient method’s operation and enhanced the consistency of its calculated results. Lastly, we employed the combined impedance method and the transient method for the high-power measurement of PZT-5H piezoelectric ceramics and obtained high-power material parameters, including $k_{31}$, $d_{31}$, $s_{11}^{E}$ and $Q_{m}$. The results from both methods indicated that with increasing power, the $k_{31}$, $d_{31}$, and $s_{11}^{E}$ of the PZT-5H showed an increasing trend, while the $Q_{m}$ decayed rapidly first and then stabilized at a fixed level. It is found that, in addition to the increase in power, the rise in temperature also leads to a decrease in $f_{s}$ and $f_{p}$. Moreover, this effect becomes more pronounced as the self-heating temperature increases. Compared to the transient method, where temperature rise is negligible, an increase in temperature significantly amplifies its effects on the $k_{31}$, $s_{11}^{E}$, and $Q_{m}$. However, the impact of temperature on the $Q_{m}$ is not significant. In the combined impedance method, even as power increases and the temperature rises, the $Q_{m}$ still remains stable. This experiment decouples the effects of temperature and power, providing insights into the design and cooling of high-power piezoelectric devices.

## Data Availability

Data are contained within the article.

## Appendix A

**Table A1:** The material properties of PZT-5H.

Parameter (PZT-5H)	Symbol	Value	Unit
Density	ρ	7600	$kg/m^{3}$
Acoustic Velocity	-	3000	m/s
Curie Temperature	$T_{c}$	220	°C
Young Modulus	*E*	56	GPa
Poisson Ratio	υ	0.36	-

Figure A1 shows an example where the vibrator was excited by a 5 V sinusoidal pulse signal. The $V_{0}$ subsequently decayed after the circuit shortening at t = 0. After the Fourier transform, the $f_{s\;}$ corresponding to the 5 V from the decay curve can be found in the frequency domain. The fitting of the decay curves (t>0) can provide the coefficient β corresponding to the 5 V.
